# Evaluating the efficacy of point-of-use water filtration units in Fiji

**DOI:** 10.1186/s41182-019-0175-4

**Published:** 2019-08-07

**Authors:** Nathan Tintle, Adam Heynen, Kristin Van De Griend, Rachel Ulrich, Matthew Ojo, Emma Boven, Sarah Brokus, Randall Wade, Aaron A. Best

**Affiliations:** 1 Department of Mathematics and Statistics, Dordt University, 498 4th Ave NE, Sioux Center, IA 51250 USA; 2 Department of Statistics, Montana State University, Bozeman, MT USA; 3 Department of Biology, Hope College, Holland, MI USA

**Keywords:** Diarrhea, Fiji, Low- and middle-income country, Water treatment, Filtration

## Abstract

**Background:**

To develop and evaluate a strategy for reducing the prevalence and impact of waterborne disease, a water quality intervention was developed for Fiji by Give Clean Water, Inc. in partnership with the Fiji Ministry of Health. Residents were provided and trained on how to use a Sawyer® PointONE™ filter, while also being taught proper handwashing techniques. At the time of the filter installation, all households were surveyed inquiring about the prior 2- to 4-week period. Households were measured a second time between 19 and 225 days later (mean = 66 days).

**Results:**

To date, five economic and health outcomes have been tracked on 503 households to evaluate the efficacy of the intervention. When comparing baseline to follow-up among the 503 households, the 2-week diarrhea prevalence decreased in households from 17.5% at baseline to 1.8% at follow-up. Also, the 2-week prevalence of severe diarrhea decreased per household from 9.7% at baseline to 0.6% at follow-up. Finally, monthly diarrhea-related medical costs reduced by an average of Fijian (FJ) $3.54 per person, and monthly water expenses reduced by FJ $0.63 per person. All estimated values are obtained from general linear and logistic mixed-effect models, which adjusted for location, season, time to follow-up, household size, water source, and respondent changing. Changes in economic and health outcomes from installation to follow-up were statistically significant (*p* < 0.05) in all cases, in both unadjusted and adjusted models.

**Conclusions:**

The installation of water filters shows promise for the reduction of diarrhea prevalence in Fiji, as well as the reduction of diarrhea-related medical costs and water expenses. Future work entails evaluation in other countries and contexts, long-term health monitoring, and comparison to alternative water quality interventions.

## Background

In low- and middle- income countries, over a quarter of a million lives lost to diarrhea could be saved by teaching people about hand hygiene and half a million lives lost to diarrhea could be saved by providing clean drinking water [1]. A major source of diarrhea is fecal pathogens via fecal-oral transmission [2, 3]. Fecal contamination is a global issue, as an estimated 26% of the global population uses a water source with 1 or more fecal indicator bacteria present in 100 milliliters of water [4]. In addition, 81% of the global population does not wash their hands with soap after possible fecal contamination [5]. Therefore, an opportunity for change is possible through preventative means by stopping the transmission of diarrhea pathogens by drinking clean water and using proper hand hygiene [3, 6].

In Fiji, an estimated 6.4% of deaths of children ages 1 month to 5 years was a result of diarrhea [7]. Furthermore, a 2009 study in Fiji’s largest hospital found that 39% of children under the age of 5 who were admitted for diarrhea tested positively for rotavirus [8]. Beginning in 2012, one major prevention initiative that Fiji has taken to counteract this problem is vaccination of children for the fecal-oral pathogen rotavirus [3, 9]. As the country moves forward in the prevention of diarrhea with vaccination against rotavirus, the country of Fiji can continue to make other adjustments to lessen the burden of diarrhea caused by non-viral agents. In efforts to address other preventative options for Fijians, Give Clean Water, Inc. [10], in partnership with the Fiji Ministry of Health, has been providing point-of-use water filtration systems (Sawyer® PointONE™) to the people of Fiji along with instructing them on handwashing since 2008.

The Sawyer® PointONE™ is a hollow membrane, point-of-use water filter with the function of removing bacteria and protozoa. Laboratory tests with the Sawyer® PointONE™ water filter suggest it aligns with the United States Environmental Protection Agency standard for bacteria and protozoa removal [11]. Results from previous studies regarding health outcomes, water contamination, and implementation are mixed. A cluster-randomized study of households evaluated diarrhea prevalence in children under the age of 5 years by comparing four interventions: a control, a training on healthy living (WASH training), providing a Sawyer® PointONE™ filter, and combination of training and the filter. Over a 3-month period, a significant decrease in diarrhea prevalence for children under the age of 5 was recorded when comparing the filter arm or the combination of the filter and training on WASH to the control arm [11]. However, a study of Sawyer® PointONE™ filters that had been distributed in Fiji 1 to 3 years prior to the study identified bacterial contamination in 17 out of 24 samples taken directly from filters and 28 out of 37 samples taken from stored filtered water as determined by sulfide paper testing [12]. Also, the study noted in an accompanying survey that only 30% of participants used the filter every time they drank water, and broken filters were observed in 22% of surveyed households [12]. Another study evaluated six Sawyer® PointONE™ filters that had been used for 23 months in Honduras. In this analysis, sterile water inserted into the filters exited contaminated with bacteria [13]. Further examination by chemical analysis and visual magnification showed evidence that filter pores had been covered by debris [13]. However, the study has received scrutiny including (a) poor pre-analysis filter storage conditions, (b) crude filter cartridge entry, (c) small sample size, and (d) inconsistencies in article figures [14]. In a similar study, the microbiological removal by the filter was compared in laboratory and field settings. The laboratory filter and the new field filters removed on average greater than or equal to 99.5% of *E. coli* or total coliform in non-filtered water; on the other hand, filters that had been in the field for 1 to 3 years had only an 89.5% reduction of *E. coli* and 67.9% reduction of total coliforms [15].

Given some of these discrepancies in the published literature about the effectiveness of Sawyer® PointONE™ filters at eliminating bacteria and reducing the risk of waterborne disease (e.g., diarrhea), this study aims to provide clarity by evaluating the change in diarrhea risk over time after filter use and training in handwashing hygiene in Fiji. Specifically, the study analyzes the change in the number of people ill with diarrhea in Fiji after the intervention. Second, we explore how this risk changes for both the number of people with diarrhea as well as the severity of diarrhea, as measured by impact on work/school attendance. Finally, we also report on the economic impact of the intervention through savings in medical expenses and water costs.

## Methods

### Data collection

Between March 2016 and December 2017, approximately 1463 households in Fiji were provided a free filter by staff members or volunteers for the charity *Give Clean Water*. Filters were donated by Sawyer® to *Give Clean Water*. The intervention proceeded village to village, with initial filters and training primarily occurring on the main island of Viti Levu. Future installation and training will expand to surrounding islands, with a goal to provide all Fijian people clean water by 2020.

During the initial visit, an adult in each household was taught the proper use and cleaning of the Sawyer® PointONE™ filter, along with basic handwashing instructions. The handwashing instructions to the adults in the household was “Many sicknesses can be avoided by simply washing your hands with soap and water. Wet your hands, apply soap, rub hands together for 15 seconds, rinse, dry with a clean towel or air dry”. In addition, the adults in each household were instructed to wash their hands before filter use and clean the filter bucket regularly.

At the time of filter installation, the adult being trained provided answers to a brief (~ 10–20 questions, depending on family size) questionnaire inquiring about basic health outcomes and general demographic information. Fourteen-hundred and sixty-three villages and households received filters between March 2016 and December 2017, with 503 households receiving qualifying follow-up visits between 19 and 225 days later, in which data on all five primary measurements of filter efficacy (see the “Measures” section for description) and follow-up demographic information was obtained. To ensure proper understanding of filter instructions and survey questions, a Fijian translator was present during the process. The Dordt College IRB approved this project.

### Filter description and use

**Fig. 1:**
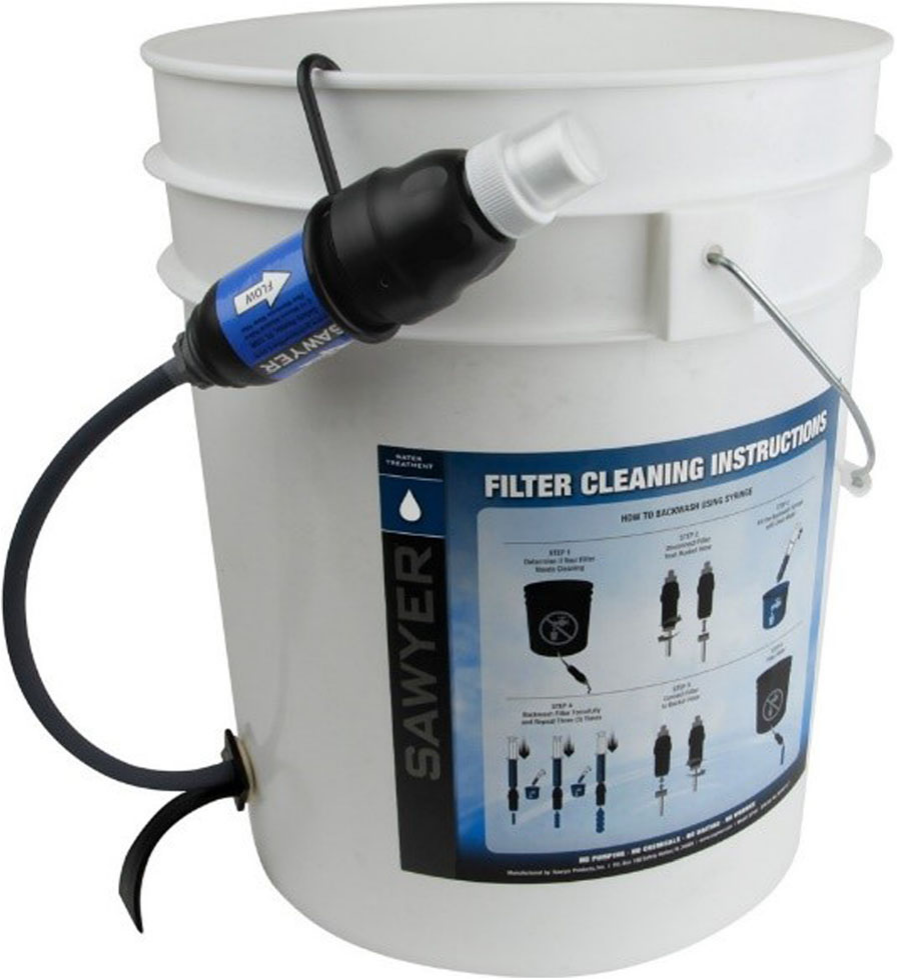
An example of the Sawyer® PointONE™ bucket filter system used in the study. The Sawyer® PointONETM bucket filter system consists of a 5-gallon bucket and filter attached with a hose. Water drains by gravity from the bucket, through the filter, and into a clean drinking water container

The Sawyer® PointONE™ filter (Fig. 1) that was distributed uses hollow fiber membranes with micro pores (0.1 microns) to ensure that bacteria and protozoa are filtered out of the water. Source water is filtered via a gravity fed bucket filter system into a clean container, removing bacteria, protozoa, and suspended particulates. The cleaning of the filter involves backflushing after each use. In addition to training an adult in each household, a printed copy of use and cleaning instructions were provided via a sticker on each bucket filter system.

### Measures

Our analysis focuses on five primary health and economic indicators. The primary variable of interest is the number of people in the household with diarrhea during the 2 weeks prior to survey administration. Secondary variables include diarrhea severity (as measured by work days missed or school days missed within the last 2 weeks) and economic impact (as the amount of money spent on medical expenses due to diarrhea and amount of money spent on water per month). In addition to these key outcomes, a variety of other household and demographic information was collected. The households revisited were compared to initial visit by matching either the household ID or the barcode ID (an identification number on the filter).

### Statistical analysis

All statistical analyses were conducted in R [16], an open-source and widely used statistical analysis program. Generalized linear mixed effects models were used to account for the repeated measures nature of the data and allow for the statistical adjustment of both fixed, random, and time-varying covariates. A binomial link (logistic regression) was used for all dichotomous response variables, with a standard link function (linear regression) for continuous response variables. Response variables were modeled in both unadjusted and adjusted models. The significance level for all tests results was 0.05.

## Results

### Sample characteristics

Table 1 provides information on the sample of the 503 households. The households come from three primary townships: Nadi, Raki Raki, and Sigatoka. Households receiving the intervention obtained water from a variety of sources including both treated (defined as government-supplied water treated by processes such as chlorination) and untreated sources, according to self-report with boreholes (e.g., a narrow shaft bored into the ground) and catchment (e.g., having rain gutters to move rainwater to a catchment tank). Small changes in water source occurred over time, with the most noticeable difference being an increase of approximately 73 households obtaining water from a treated source. While data collection proceeded year-round, follow-up visits were nearly four times as likely to be collected during the dry season as the rainy season. Thus, it is possible that the houses changing water source from baseline to follow-up is due to water sources becoming unavailable during the dry season. Households had an average of slightly over 3 adults and 1.5 children living in them with little change observed from baseline to follow-up. Time to follow-up varied greatly with an average of 63.8 days (min = 19 days; max = 225 days).

**Table 1:** Characteristics of the sample

Characteristic	Baseline	Follow-up	Significance of change^1^
Town			
Nadi	219	219	*p* = 1.0
Raki Raki	57	57	*p* = 1.0
Sigatoka	227	227	*p* = 1.0
Water source			
Borehole	128	121	*p* = 0.28
Catchment	99	99	*p* = 1.0
River/creek	88	19	*p* < 0.0001
Tap (treated)	72	145	*p* < 0.0001
Tap (untreated)	74	86	*p* = 0.30
Well	42	33	*p* = 0.049
Season			
Rainy (Dec–Apr)	213	113	*p* < 0.0001
Dry	290	390	*p* < 0.0001
Household size			
Adults	3.17 (1.54)	3.06 (1.54)	*p* = 0.07
Children	1.62 (1.60)	1.62 (1.71)	*p* = 0.90
Days between measurements	Mean = 63.8 (60.57)		
	Min =19 days; max = 225 days		

^1^*p* values from McNemar’s test or a paired *t* test depending on whether the data was quantitative or binary

### Change in diarrhea risk over time

**Fig 2:**
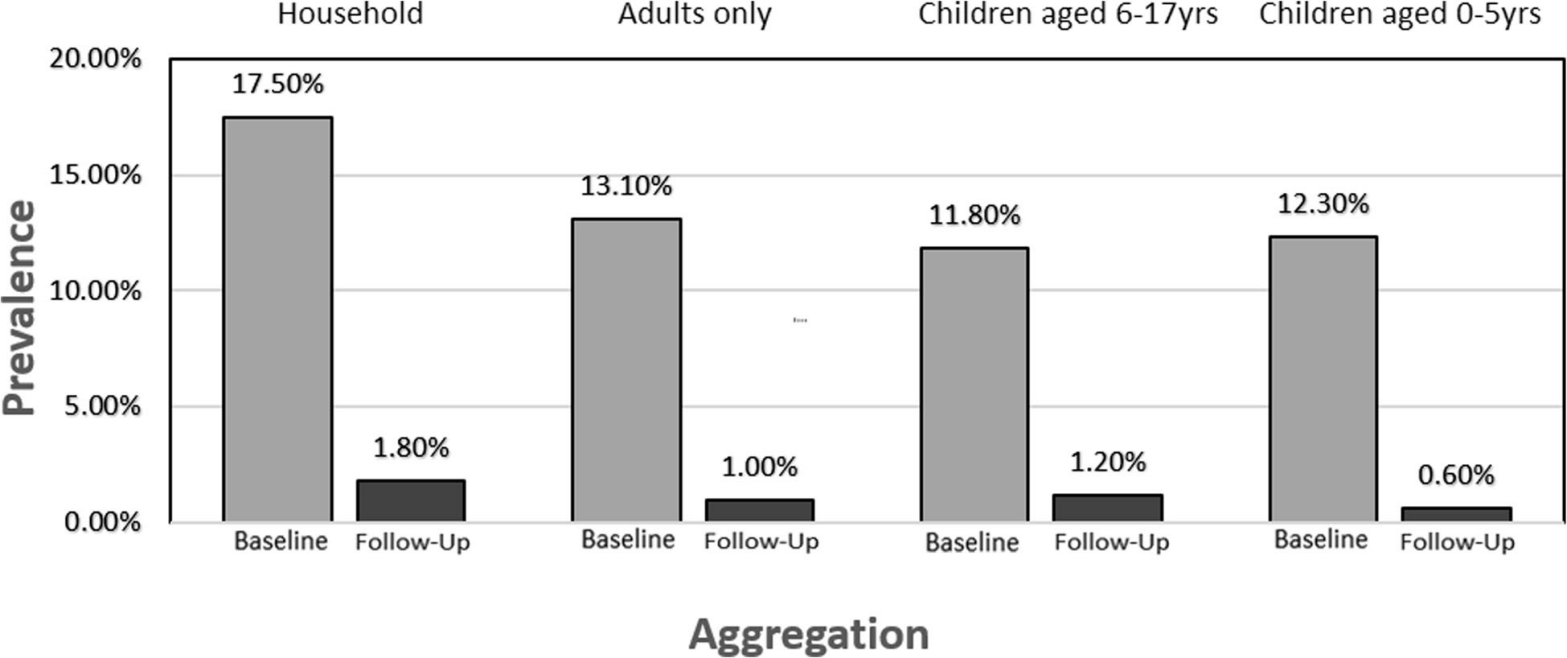
Diarrhea prevalence by age group at baseline and follow-up. Diarrhea was prevalent in 17.5% of households sampled within the 2-week period prior to intervention (baseline) while decreasing to 1.8% of households within the 2-week period prior to follow-up survey administration. Similar decreases in prevalence were noted in different age categories. All differences were statistically significant before and after adjusting for demographic and household covariates (see Tables 2 and 3 for details)

Diarrhea was prevalent in more than 1 out of 6 households in the sample within the 2-week period prior to intervention (see Table 2 and Fig. 2). Prevalence of diarrhea decreased to less than 2% of households (less than 1 in 50) within the 2-week period prior to follow-up survey administration. Similar results were found in prevalence when separating the households on whether the follow-up visit was less than or greater than 60 days from baseline visit (see Table 2). After adjusting for all variables in Table 1, risk estimates remained significant.

**Table 2:** Diarrhea prevalence by household at baseline and follow-up separated by days between measurements

Aggregation	Timing	Prevalence	Odds ratio (95% confidence interval (CI))	Adjusted odds ratio (95% CI)^4^
Household^1^	Baseline	17.5% (88/503)	11.6 (5.7, 23.7)***	14.8 (6.9, 32.0)***
	Follow-up	1.8% (9/503)	1.0	1.0
Households followed up within 60 days^2^	Baseline	17.3% (63/364)	10.7 (4.7, 24.0)***	14.4 (5.9, 35.1)***
	Follow-up	1.9% (7/364)	1.0	1.0
Households followed up after 60 days^3^	Baseline	18.0% (25/139)	15.0 (3.4, 66.8)***	56.3 (1.9, 1,653.5)*
	Follow-up	1.4% (2/139)	1.0	1.0

*p < 0.05; **p < 0.01; ***p < 0.001

^1^Summarized as whether anyone in the household experienced diarrhea within the previous 2 weeks

^2^Summarized as household being visited within 60 days of baseline intervention and whether anyone in the household experienced diarrhea within the previous 2 weeks

^3^Summarized as household being visited after 60 days of baseline intervention and whether anyone in the household experienced diarrhea within the previous 2 weeks

^4^Adjusted for all variables in Table 1 plus an indicator variable for whether the water source changed, an indicator variable for whether the person answering the survey questions changed, and a variable indicating any change in the number of adults in the household

Diarrhea prevalence for households has similar distributions across adults, school-aged children, and young children (see Table 3 and Fig. 2). Changes in diarrhea risk were statistically significant overall and within each age stratum. After adjusting for potential confounding variables, the risk estimates remained significant.

**Table 3:** Diarrhea prevalence by age group at baseline and follow-up

Aggregation	Timing	Prevalence	Odds ratio (95% CI)	Adjusted odds ratio (95% CI)^3^
Household adults only^1^	Baseline	13.1% (66/503)	15.0 (5.9, 38.4)***	18.6 (6.8, 51.0)***
	Follow-up	1.0% (5/503)	1.0	1.0
Household children aged 6–17 years only^2^	Baseline	11.8% (30/254)	11.2 (3.3, 38.2)***	13.6 (3.8, 49.2)***
	Follow-up	1.2% (3/254)	1.0	1.0
Household children aged 0–5 years only^2^	Baseline	12.3% (20/163)	22.7 (2.9, 178.2)**	31.8 (3.6, 281.5)**
	Follow-up	0.6% (1/163)	1.0	1.0

*p < 0.05; **p < 0.01; ***p < 0.001

^1^Summarized as whether any adults in the household experienced diarrhea within the previous 2 weeks

^2^Summarized as whether any children in the household experienced diarrhea within the previous 2 weeks

^3^Adjusted for all variables in Table 1 plus an indicator variable for whether the water source changed, an indicator variable for whether the person answering the survey questions changed, and a variable indicating any change in the number of adults in the household

### Diarrhea severity

Similar to overall diarrhea risk, when we explored the risk of severe diarrhea, as measured by being severe enough to cause the affected individual to miss work (adult) or school (school-aged child), we found a similar decreasing pattern from baseline to follow-up (see Table 4). While the overall prevalence of severe diarrhea at baseline is lower than the number of people with diarrhea (approximately 1 in 10 households affected with severe diarrhea over a 2-week period), the decrease from baseline prevalence to follow-up is still present.

**Table 4:** Severe diarrhea by age group at baseline and follow-up

Aggregation	Timing	Prevalence	Odds ratio (95% CI)	Adjusted odds ratio (95% CI)^4^
Household^1^	Baseline	9.7% (49/503)	18.0 (5.4, 59.5)***	29.5 (7.5, 115.2)***
	Follow-up	0.6% (3/503)	1.0	1.0
Adults only^2^	Baseline	7.4% (37/503)	Infinite***^5^	Infinite***^5^
	Follow-up	0.0% (0/503)	1.0	1.0
Children aged 6–17 years only^3^	Baseline	7.9% (20/254)	7.2 (2.1, 25.1)**	8.5 (2.3, 31.6)**
	Follow-up	1.2% (3/254)	1.0	1.0

*p < 0.05; **p < 0.01; ***p < 0.001

^1^Summarized as whether anyone in the household experienced diarrhea within the previous 2 weeks so that it caused them to miss work (adults) or school (school-aged child)

^2^Summarized as whether any adult in the household experienced diarrhea within the previous 2 weeks severe enough to miss work

^3^Summarized as whether any children in the household experienced diarrhea within the previous 2 weeks severe enough to miss school

^4^Adjusted for all variables in Table 1 plus an indicator variable for whether the water source changed, an indicator variable for whether the person answering the survey questions changed, and a variable indicating any change in the number of adults in the household.

^5^ Infinite odds ratios because the prevalence was 0.0% at follow-up

### Economic impact

Table 5 provides the analysis of two economic indicators related to diarrhea: medical expenses due to diarrhea and household water expenses. In both cases, substantial changes were observed moving from baseline to follow-up with estimated savings of approximately Fijian (FJ) $3.50 in medical costs and FJ $0.75 in water expenses, per person, per month on average. These results remained statistically significant even after adjusting for other variables.

**Table 5:** Economic impact (FJ$) of diarrhea by age group at baseline and follow-up

Aggregation	Timing	Mean (SD)	Unadjusted difference (95% CI)	Adjusted difference (95% CI)^2^
Household medical expenses due to diarrhea per month per person^1^	Baseline	$3.84 ($11.73)	$3.54 ($2.47, $4.61)***	$4.40 ($3.29, $5.51)***
	Follow-up	$0.30 ($2.76)		
Household water expenses per month per person^1^	Baseline	$0.78 ($2.88)	$0.63 ($0.35, $0.92)***	$0.74 ($0.46, $1.03)***
	Follow-up	$0.15 ($1.39)		

*p < 0.05; **p < 0.01; ***p < 0.001

^1^Amount reported by household per month divided by the reported number of people in a household

^2^Adjusted for all variables in Table 1 plus an indicator variable for whether the water source changed, an indicator variable for whether the person answering the survey questions changed, and a variable indicating any change in the number of adults in the household

## Discussion

The importance of providing effective, sustainable, and economically practical water filtration systems cannot be understated. However, additional work is needed to understand which filters/systems are effective in the field due to numerous challenges that come with real people using real products in uncontrolled settings over lengthy periods of time. This analysis suggests that the combination of providing a Sawyer® PointONE™ filtration system and training on handwashing hygiene (the intervention) provided substantial health and economic benefits to a sample of individuals in the country of Fiji.

The decrease from baseline to follow-up in diarrhea prevalence, severe diarrhea, and economic impact shows the benefit of the filter and handwashing hygiene intervention. To provide a more specific context of the economic impact, the current minimum wage is FJ $2.68 [17]. The amount of savings from water and medical expenses per Fijian per month, provided intervention, totals to be FJ $ 4.17 per month per person. This is equivalent to approximately 1.56 h of labor for a Fijian minimum wage worker.

Several strengths and limitations of this analysis are worth noting. First, we used a general linear modeling approach in order to account for both fixed and time-varying covariates in the pre-post study design, allowing us to address confounding variables both in the design (matched pairs) and analysis (time-varying covariates). Second, we note that while following-up within households ensures that each individual household serves as its own control, additional follow-ups are needed of the full, unbiased set of households in which filters have been installed, and with increased sample sizes over varying lengths of time. While there is no evidence of changing efficacy over time in this analysis (longest time to follow-up was 255 days), additional data are needed over multiple years to conclusively state the long-term efficacy and viability of the proposed Sawyer® PointONE™ and handwashing hygiene intervention. While cleaning is simple (a simple backwash procedure to be completed after each filter use), additional information on long-term compliance/utilization of the filters over time and backwashing technique is needed. Third, while this study, combined with prior laboratory testing, provides compelling evidence of the efficacy of the intervention to remove waterborne bacteria, this study only focuses on self-reported health outcomes and does not separate any unique effects of handwashing instruction from filter use. However, prior studies have found a similar impact from filters alone compared to filters and handwashing instruction [18]. Importantly, we note that the handwashing intervention was quite minimal and not a major part of this intervention. Finally, and importantly, the government’s vaccination program for rotavirus is likely contributing to the low prevalence of diarrhea in children at baseline and may impact the generalizability of the findings to other countries which do not vaccinate.

## Conclusions

Overall, the intervention of water filtration and handwashing instruction showed great promise in reducing diarrhea and other health factors and in improving economic conditions for households in Fiji. The use of a large sample size sheds further light on the effectiveness of this water filter intervention, and this study’s economic analysis adds a dimension unaddressed in previous research. Further work is needed to do water sample testing from the filters in the field to ensure short and long-term efficacy. Finally, we note that conclusive causal evidence of the impact of the intervention will only be possible in a randomized trial.

## Data Availability

The datasets used and/or analyzed during the current study are available from the corresponding author on reasonable request.

## Abbreviations

FJ$: Fiji dollars
WASH: water, sanitation, and hygiene
